# A preliminary study on the use of noninvasive hemodynamic monitoring with the Nexfin monitor in critically ill patients

**DOI:** 10.1186/cc10834

**Published:** 2012-03-20

**Authors:** M Peetermans, W Verlinden, J Jacobs, A Verrijcken, S Pilate, N Van Regenmortel, I De laet, K Schoonheydt, H Dits, ML Malbrain

**Affiliations:** 1 ZNA Stuivenberg, Antwerp, Belgium

## Introduction

Noninvasive hemodynamic monitoring may become a new tool in the ICU armamentarium. The Nexfin monitor (BMEYE, Amsterdam, the Netherlands) enables continuous noninvasive analysis of the finger blood pressure waveform using an inflatable finger cuff, a technology based on the volume-clamp principle of Penaz in combination with the physical criteria of Wesseling. The aim of the present study was to validate the Nexfin in a mixed population of medical ICU patients and to look for a pattern recognition that may be linked with outcome.

## Methods

A prospective study in 40 patients admitted to the medical ICU (17 patients mechanically ventilated, M/F ratio 1/1). Age 63.5 ± 16.7, BMI 26.4 ± 5.4, APACHE II score 20.8 ± 9.5, SAPS II 45.9 ± 18.9, SOFA score 7.2 ± 4.2. For all patients, simultaneous recording of arterial pressure by radial line (*n *= 46), by PiCCO monitor (*n *= 15) or by NIBP measurement with arm cuff (*n *= 17) was compared with noninvasive hemodynamic parameters obtained with the Nexfin monitor. Statistical analysis was performed with Student's *t *test, Pearson correlation and Bland-Altman analysis.

## Results

A total of 69 measurements in 40 patients were performed. In three patients measurement with the Nexfin was not possible. For CO (26 paired measurements), values were 6.4 ± 2.1 l/minute (range 3.3 to 12). The Pearson correlation coefficient comparing Nexfin-CO with reference CO showed a good correlation (*R*^2 ^= 0.5). Bland-Altman analysis comparing both CO techniques revealed a mean bias ±2SD (LA) of 0.7 ± 3.9 l/minute (58.3% error). The MAP was 84.6 ± 17.7 mmHg (57.5 to 131.5) and values obtained with the Nexfin correlated well with the reference method (PiCCO in eight; radial line in 43) with an *R*^2 ^of 0.75. Bland-Altman analysis comparing both MAP techniques revealed a mean bias ±2SD (LA) of 0.2 ± 19.7 mmHg (23.3% error). However, Nexfin-MAP did not correlate well with NIBP (*R*^2 ^= 0.1). The nine patients that died in the ICU had higher APACHE II (*P *= 0.07), SAPS II (*P *= 0.07) and SOFA (*P *= 0.01) scores and significantly lower MAP (*P *= 0.028) and lower dp/dtmax (*P *= 0.029), a marker for contractility. There were no outcome differences with regard to subgroup analysis in patients with either low or high CO or SVR.

## Conclusion

The preliminary results of this ongoing prospective trial indicate that in unstable critically ill patients CO and MAP can be monitored noninvasively with the Nexfin. The exact patient population for this technology has yet to be defined and more patients are probably needed for pattern recognition, although the results indicate that low MAP and dp/dtmax are associated with poor outcome.

